# Design and Optimization of a Self-Protected Thin Film c-Si Solar Cell against Reverse Bias

**DOI:** 10.3390/ma16062511

**Published:** 2023-03-21

**Authors:** Omar M. Saif, Abdelhalim Zekry, Ahmed Shaker, Mohammed Abouelatta, Tarek I. Alanazi, Ahmed Saeed

**Affiliations:** 1 Department of Electronics and Communications, Faculty of Engineering, Ain Shams University, Cairo 11566, Egypt; 2 Engineering Physics and Mathematics Department, Faculty of Engineering, Ain Shams University, Cairo 11566, Egypt; 3 Department of Physics, College of Science, Northern Border University, Arar 73222, Saudi Arabia; 4 Electrical Engineering Department, Future University in Egypt, Cairo 11835, Egypt

**Keywords:** self-protection, c-Si solar cell, reverse bias, backward diode, antireflection coating

## Abstract

Current mismatch due to solar cell failure or partial shading of solar panels may cause a reverse biasing of solar cells inside a photovoltaic (PV) module. The reverse-biased cells consume power instead of generating it, resulting in hot spots. To protect the solar cell against the reverse current, we introduce a novel design of a self-protected thin-film crystalline silicon (c-Si) solar cell using TCAD simulation. The proposed device achieves two distinct functions where it acts as a regular solar cell at forward bias while it performs as a backward diode upon reverse biasing. The ON-state voltage (*V_ON_*) of the backward equivalent diode is found to be 0.062 V, which is lower than the value for the Schottky diode usually used as a protective element in a string of solar cells. Furthermore, enhancement techniques to improve the electrical and optical characteristics of the self-protected device are investigated. The proposed solar cell is enhanced by optimizing different design parameters, such as the doping concentration and the layers’ thicknesses. The enhanced cell structure shows an improvement in the short-circuit current density (*J_SC_)* and the open-circuit voltage (*V_OC_*), and thus an increased power conversion efficiency (PCE) while the *V_ON_* is increased due to an increase of the *J_SC_*. Moreover, the simulation results depict that, by the introduction of an antireflection coating (ARC) layer, the external quantum efficiency (EQE) is enhanced and the PCE is boosted to 22.43%. Although the inclusion of ARC results in increasing *V_ON_*, it is still lower than the value of *V_ON_* for the Schottky diode encountered in current protection technology.

## 1. Introduction

PV technologies provide clean and reliable means to meet the ever-increasing demand for energy. For several decades, silicon solar cells have represented the dominant technology in PV industries [1,2,3] thanks to their non-toxic properties [1,4]. Additionally, the energy band gap of silicon is 1.12 eV, which corresponds to an absorption cut-off wavelength of about 1160 nm [5]. Recently, the production of thin-film c-Si cells with lower than 50-μm-thick c-Si wafers has been endeavored [6,7,8,9,10,11,12,13]. These thin-film c-Si cells have the advantage of being extremely cost-competitive and can be fabricated based on the traditional processes of thick c-Si solar cells [13,14]. Yet, planar c-Si solar cells with a low thickness of 50-μm suffer from a light absorption loss because of the mismatch in the refractive index between the c-Si and the air [6,8,13]. In order to create extremely effective thin-film c-Si solar cells, it is important to significantly thin the c-Si wafers and use recently discovered light-trapping methods to absorb incident solar radiation in thin-film c-Si devices. These techniques include using an antireflective coating, as well as applying nano- and micro-structures, including nanowires [15,16], nanocones [8,17], and textured structures [18,19,20,21]. Further, thin-film c-Si solar cells may be flexible [10,11], thereby expanding the variety of their applications. Consequently, the development of thin-film c-Si solar cells will be a crucial and novel technological trend that can have several profound effects. Although the reported efficiencies of thin-film silicon-based cells still lag behind those of conventional first-generation silicon cells, more developments are being conducted in order to improve their PCE to be competitive in certain applications that involve flexibility and low weight.

Usually, the PV module utilizes strings of solar cells attached in series so that the required output power can be generated [22]. These series-connected cells make the PV system highly sensitive to the current mismatch conditions. The current mismatch may appear because of partial shading conditions or malfunction of some c-Si cells. Therefore, the defective or shaded cells become reverse-biased and act as loads to the other illuminated strings, resulting in power dissipation. This dissipation causes an overheat of the module or creates hot spots [23]. Consequently, a current mismatch not only affects the efficiency of the individual cells, but also unfavorably influences the reliability of the overall PV module [24,25].

Many research studies have been conducted to overcome the mismatch conditions. However, more efforts are still required to obtain extra efficient solutions. One possible solution to the current mismatch was to integrate a bypass diode in parallel with every single cell [26,27,28,29]. This methodology was not beneficial for the PV module because it involved higher costs [30]. The primary protection approach utilized recently against reverse bias situations is a passive bypass Schottky diode that is arranged in parallel with a string of cells [31,32,33]. Additionally, the latest investigations emphasize alleviating the influence of localized overheating or hot spots and improving the module reliability by adapting the bypass (protection) circuit [34,35,36,37,38,39]. However, the protection circuits or the bypass Schottky diodes can result in consuming more power and complicating the design, as well as raising the module costs.

This research study introduces a state-of-art design that can serve as a self-protected thin-film c-Si solar cell. The technique is based on proposing a P^+^ reverse-conducting layer (RCL) to be added as an extra layer just below the emitter, which can be introduced before the formation of the emitter. The study investigates the electrical and optical characteristics of both forward and reverse biasing conditions. At the forward bias condition, the solar cell operates as a regular and efficient cell, while it functions as a backward diode under the reverse bias condition. Hence, there is no requirement to use bypass diodes or protection circuits. In addition, improvement techniques are applied to optimize the performance of the proposed device. The study is performed by a numerical TCAD simulation, which is extremely helpful to realize the key physical behaviors, thereby reducing development time and cost.

The paper is organized as follows. Section 2 presents the basic device structure of the novel device and the physical models used in the device simulation. Additionally, Silvaco ATLAS tool calibration vs. measurements is introduced. Section 3 presents the electrical and optical characteristics of the proposed device and compares the simulation results for such a device with those of the device without RCL (normal structure). In addition, the *V_ON_* at the reverse bias of the proposed device is theoretically investigated. Then, in Section 4, the enhancement of the self-protected thin film c-Si solar cell is numerically accomplished by optimizing some design parameters. Further, the incorporation of the ARC layers on the proposed cell is studied in Section 5 to explore its impact on the performance, especially the ON-state voltage. Finally, the basic findings and conclusions of this work are summarized in Section 6.

## 2. Simulation Methodology and Device Structure

In this section, we present the structure of the basic single-junction self-protected thin-film c-Si solar cell. Firstly, the essential physical models for the solar cell design are reviewed. Then, we provide a calibrated thin-film structure that is based on an experimental cell, along with its available parameters. Finally, the device structure of our proposed cell is presented.

### 2.1. Physical Models and Material Parameters

All the numerical simulations are performed by employing Silvaco TCAD device simulator. The electrical simulation is achieved by utilizing the drift-diffusion model. Poisson and carrier continuity equations are concurrently solved regarding both charge carriers (electrons and holes). Further, Shockley–Read–Hall recombination with doping dependence model (CONSRH) is activated. In addition, as Auger recombination is significant for concentrations around 1 × 10^19^ cm^−3^ or higher for Si [40], it is included in the simulation, as the structure has some heavily doped regions (>1 × 10^18^ cm^−3^) [41]. Although radiative recombination has a minor effect on the performance of a practical silicon solar cell, it is crucial for basic modeling and for the optoelectronic characterization of silicon solar cells. Modeling of radiative recombination is commonly achieved by enabling the optical radiative recombination (OPTR) model. The bandgap narrowing effect is activated in ATLAS device simulator by means of the BGN model. Concerning the mobility model, the FLDMOB model activates the electric field-dependent mobility, while the CONMOB model identifies the concentration-dependent mobility. This latter model is based on previous experimental results for Si at 300 K. Moreover, to model any possible tunneling currents, the non-local band-to-band (BTB) tunneling model (BBT.NONLOCAL) is applied [42] to trace any possible tunneling due to high doping of the RCL. Regarding the optical simulations, the transfer matrix method (TMM) is used to compute the intensity distribution and photogeneration profiles inside the device under the illumination of the AM1.5G spectrum. The refractive (*n*) and extinction coefficient (*k*) for Si that depend on wavelength are taken from the Sopra database [43].

The main required material parameters for tool calibration and design of the self-protected c-Si solar cell are summarized as follows. The energy bandgap is *E_g_* = 1.12 eV the electron affinity is *χ* = 4.05 eV, and the dielectric permittivity is *ε_r_* = 11.7. In addition, the conduction band and valence band density of states are *N_C_* = 2.8 × 10^19^ cm^−3^ and *N_V_* = 1.04 × 10^19^ cm^−3^, respectively [42].

### 2.2. Silvaco ATLAS TCAD Tool Calibration

In this subsection, we provide a calibration step based on modeling a thin-film structure using its available physical parameters introduced in the literature [13]. The fabricated structure basically consists of three main layers: emitter, base, and back surface field (BSF) layers. The thin-film device has a base thickness of 20 μm [13]. All other technological and physical parameters, such as doping concentrations and thicknesses of the emitter and BSF, were fine-tuned in such a way as to match the simulated device performance against the corresponding experimental data. For the calibration process, the doping concentrations of the emitter, the base, and the BSF were selected to be 5 × 10^18^ cm^−3^, 5 × 10^17^ cm^−3^, and 5 × 10^18^ cm^−3^, respectively. Meanwhile, the thicknesses for both the emitter and the BSF layer equaled 100 nm and 1000 nm, respectively. Further, the ARC layers were applied in the calibration process. After running the ATLAS device simulator, we obtained the simulated thin-film c-Si solar cell performance parameters, such as the *V_OC_*, the *J_SC_*, the fill factor *FF*, and the PCE. For validation purposes of the simulation results alongside the physical parameters, the simulation results are compared with the corresponding experimental values reported in [13]. As can be inferred from Table 1, the simulation results closely match the experimental results.

**Figure 1:**
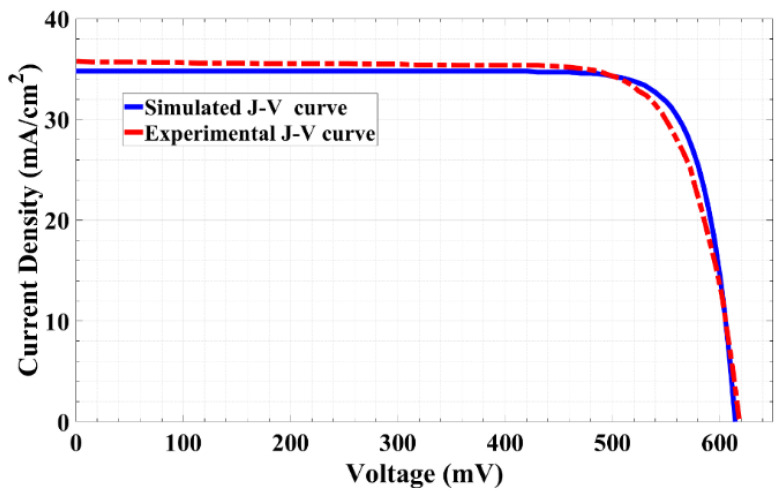
Simulated and experimental curves of the illuminated *J-V* characteristics.

Furthermore, the illuminated *J-V* characteristics were simulated, and the results are plotted together with the experimental results in Figure 1. It is clear from Figure 1 that the simulation results agree well with the experimental data, which verifies the accuracy of the model, and the Silvaco ATLAS simulation is well-calibrated regarding this type of solar cell.

**Table 1:** TCAD results for the calibrated cell in comparison with experimental data.

	*V_OC_* [V]	*J_SC_* [mA/cm^2^]	*FF* [%]	*PCE* [%]
Experimental Results [13]	0.6180	35.30	78.30	17.300
TCAD Calibration Results	0.6042	34.86	82.40	17.416

### 2.3. Device Structure

**Figure 2:**
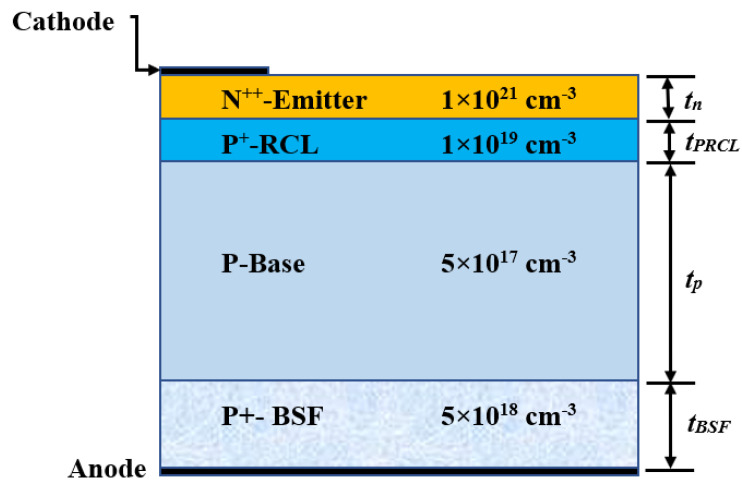
Representative figure of the proposed self-protected thin-film Si cell including a reverse conducting layer.

The construction of the single-junction self-protected thin-film c-Si cell is based on the calibrated solar cell [13] with some modifications. The main configuration of the proposed cell is displayed in Figure 2. It is an N^++^/P^+^/P/P^+^ configuration that has a full back metallization and partially front contact. The normal parameters of the proposed cell are as follows. The p-layer substrate thickness is *t_p_* = 20 µm, the thickness of the reverse-conducting p-layer is *t_PRCL_* = 12 nm, and the emitter layer (n^++^-layer) thickness is *t_n_* = 100 nm. The reverse-conducting p-layer thickness of 12 nm was chosen as an initial value, which was then investigated and optimized. The doping density of the p-layer substrate was chosen to be 1 × 10^17^ cm^−3^ [44], and the initial doping density of the P^+^ RCL was 1 × 10^19^ cm^−3^, while the emitter was selected to be heavily doped [45]. The primary distinction between our proposed structure and the calibrated structure is that our proposed structure is strongly reliant on high-doped RCL. The RCL performs a crucial role in establishing the function of the solar cell under investigation when the cell works in forward bias and small reverse bias conditions, as will be demonstrated in the following subsections.

Here, in our structure, the doping concentration of the emitter was selected to be higher than its value in the calibrated structure. The reason behind this will be discussed further in the upcoming section. This makes the *V_OC_* of our proposed structure greater than that of the calibrated structure. Also, our device was initially constructed without any ARC layers to provide a transparent investigation about the inclusion of the RCL without incorporating other effects. However, the impact of the ARC was then explored to test its role in the device performance and how it can modify *V_ON_*. Since no ARC layers were utilized in the first design stage, it was predicted that the *J_SC_* will be lower than that of the calibration. A high-doped BSF layer of a density of 5 × 10^18^ cm^−3^ and thickness *t_BSF_* of 1000 nm [46,47,48] was implemented. An electric field that forms at the interface between the base and the BSF layer creates a barrier to the electron flow to the back surface resulting in back surface passivation [49]. This has a direct effect on increasing the *J_SC_* and *V_OC_* for the presented cell [50], thereby boosting its PCE.

## 3. Results and Discussion

**Figure 3:**
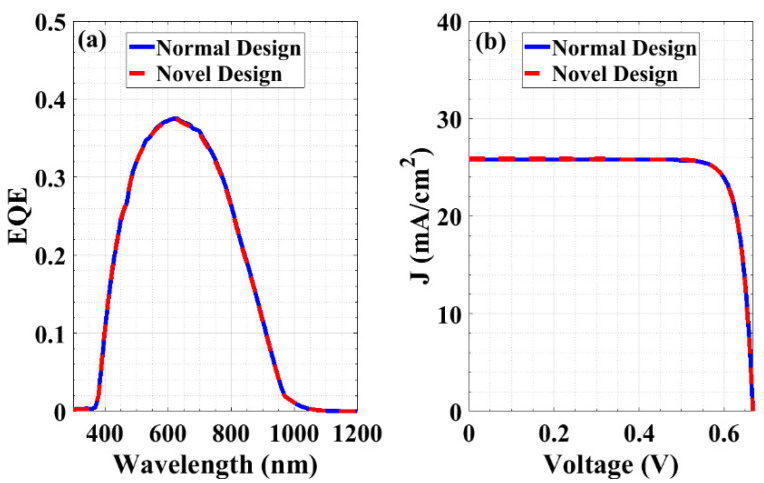
Optoelectronic characteristics of both the novel and normal designs under illumination (**a**) EQE and (**b**) *J-V* characteristics.

**Figure 4:**
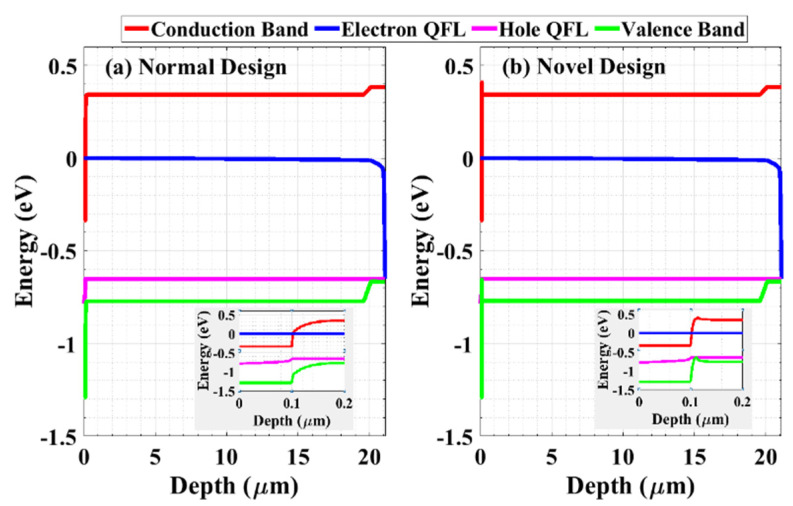
The energy band diagram at *V_OC_* for (**a**) the normal design and (**b**) the novel design. The insets show a zoomed-in depth to investigate the behavior of the quasi-Fermi levels before and after applying the RCL.

As mentioned herein, the proposed cell incorporates a P^+^ RCL between the emitter and the base. To signify its effect and the difference between the proposed cell with the RCL (novel design) and the conventional structure without the RCL (normal design), the optical and electrical performances of both cells are demonstrated in Figure 3a,b, respectively. The optical characteristics, in terms of EQE, for both cells are almost identical, implying the same *J_SC_* for both cases. Regarding the electrical characteristics in terms of the *J-V* curves, the *V_OC_* and *J_SC_* values are nearly the same for both the novel and normal structures. This is due to the shift of the quasi-Fermi level (QFL) toward the band edges being almost the same for both cells. In order to illustrate these similarities, Figure 4 shows the energy band profile, including the electron and hole QFLs for the normal design (Figure 4a) and the proposed design (Figure 4b).

The insets of Figure 4 introduce a zoomed-in depth in order to show the similarity in the QFLs before and after introducing the RCL. This produces equal contact differences in electrostatic potential. Consequently, the efficiency and maximum power transferred for the proposed and conventional solar cells are almost identical. Thus, the RCL has no adverse influence on the electrical functioning of the cell when it is forward-biased. Table 2 lists the key PV factors for both structures under illumination.

**Figure 5:**
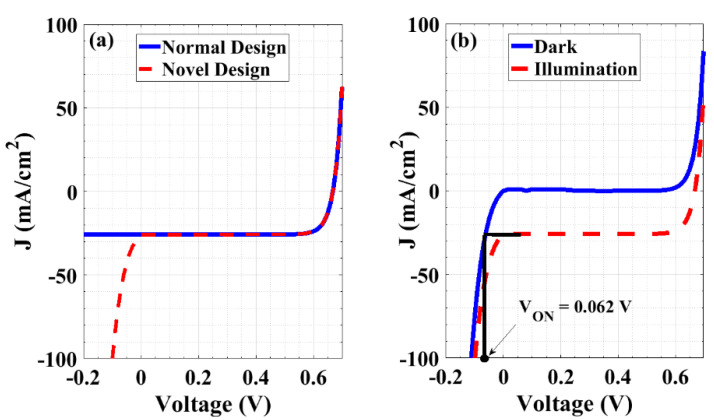
(**a**) Comparison of the electrical performance under an illumination condition for both the normal and novel structures, and (**b**) determining ON-state voltage of the proposed cell.

Figure 5a displays the performance of the devices in forward and reverse bias situations. As can be inferred from the figure, the *J-V* curve of the novel design at the reverse segment reveals a conduction mechanism that takes place at a few millivolts. This behavior indicates an efficient rectifier operation over a limited voltage range, but in the backward direction (backward diode). Notably, the most critical aspect that determines the cell performance when applying a reverse bias (case of shadowing/malfunctioning cells) is the *V_ON_* of the backward diode. The *V_ON_* is defined as the diode voltage drop at which the backward diode starts to conduct. That is, it determines how fast the response of the backward diode will be. It is determined by the projection at the x-axis of the point of intersection between the shaded reverse *J-V* curve (where the dark situation is the worst case) and the forward *J-V* curve of the unshaded cell [51,52,53]. In other words, it can be defined when the reverse short-circuit current of the shaded cell reaches the value of the short-circuit current of the unshaded cell (Figure 5b).

It will be demonstrated in the next section that the larger the short-circuit current of the unshaded cell is, the larger the ON-state voltage of the backward diode occurs. It was found that the *V_ON_* of the novel device is lower than that of the best bypass diode (Schottky diode) integrated on the PV panels [54,55], where its value is 0.35–0.45 V for a practical diode, while it was 0.062 V for our initial design. Consequently, the proposed device exhibits the capacity to rapidly reduce the influence of the reverse bias voltage, owing to malfunctioning cells or partial shading and, as a result, the capacity to avoid overheating or hot spots in a PV module.

Furthermore, the proposed design provides self-protection. This means that each cell has its own safety to reverse current by the maximum value of the short-circuit current. This is another benefit of the presented design over the bypass diode, as it protects the cell by itself, while the bypass diode protects the string of connected cells. Thus, when the operation is at reverse bias, by utilizing the proposed design, a power loss will occur because of the malfunctioning cells only. Nevertheless, by using the bypass diodes, the power loss will occur because of the string of cells. As a result, the power dissipated through the presented design in this work is lower than the power dissipated when employing bypass diodes.

## 4. Enhancement Techniques for the Proposed Cell

This section first presents the effect of the doping density and the thickness of the RCL. Secondly, the influence of the doping concentration of the emitter is studied. Finally, the impact of the doping concentration of the BSF layer on the performance of the proposed cell is presented.

### 4.1. Impact of RCL Doping and Thickness

**Figure 6:**
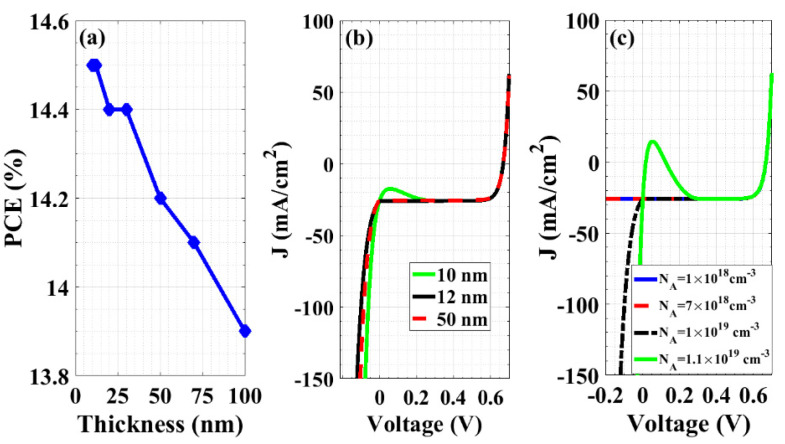
(**a**) Effect of the thickness of the RCL on the performance of the self-protected structure, (**b**) *J-V* curves for the RCL thickness of 10 nm, 12 nm, and 50 nm, and (**c**) *J-V* curves for different doping concentrations of the RCL.

Here, we study the effect of the doping density and the thickness of the RCL on cell performance. The most suitable doping density and thickness are those at which the solar cell functions with the maximum efficiency at forward bias while operating as a perfect backward diode in reverse bias. Figure 6a reveals that the best thickness between the selected range (10 nm to 100 nm) is 10 nm, for which the maximum efficiency can be obtained. However, at reverse bias and using a thickness of RCL of 10 nm, the device does not operate as an efficient backward diode, as illustrated in Figure 6b, which illustrates the *J-V* curves for three various thicknesses. Instead, the cell operates as a tunnel diode when the RCL thickness is 10 nm. Although higher thicknesses give appropriate reverse operation, the obtained PCEs at these thicknesses became lower. Therefore, we selected a thickness of 12 nm, which is the minimum thickness of the RCL that ensures a backward diode when applying a reverse bias.

**Figure 7:**
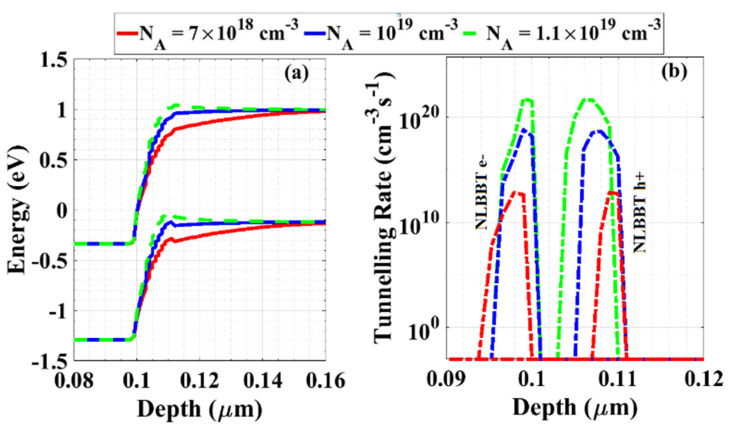
(**a**) Energy band diagram at the interface between the emitter and the RCL to show the band-to-band tunneling at different doping concentrations of RCL, and (**b**) the nonlocal band-to-band electron and hole tunneling rates.

Additionally, the next simulation considers the variation of the doping concentration in the range of 1 × 10^18^ cm^−3^ to 2 × 10^19^ cm^−3^. One can observe that the proposed cell operates at reverse bias as a backward diode when the doping concentration of the RCL is in the range of 7 × 10^18^ cm^−3^ to 1 × 10^19^ cm^−3^, while raising the doping above 1 × 10^19^ cm^−3^ makes the cell behave as a tunnel diode, as evident in Figure 6c. The behavior of tunneling that appears for high doping can be explained by plotting the energy band diagrams for three selected cases, as displayed in Figure 7a. The figure shows that upon increasing the doping to 1.1 × 10^19^ cm^−3^, the bands tend to be closer and the minimum tunneling width decreases, resulting in higher tunneling rates, as displayed in Figure 7b.

### 4.2. Impact of Emitter Doping Concentration

**Figure 8:**
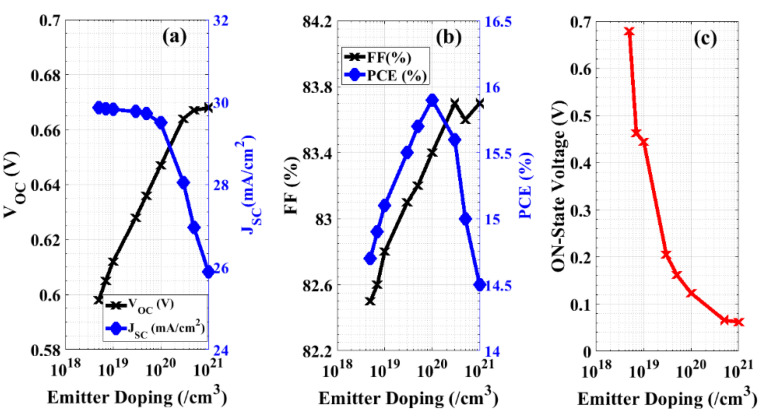
Performance of the novel cell by optimizing the doping concentration of the emitter. (**a**) Open-circuit-voltage and short-circuit current density, (**b**) fill factor and power conversion efficiency, and (**c**) ON-State voltage.

In this subsection, the impact of various doping concentrations of the emitter film on the performance of the presented device is introduced. The doping concentration is varied, starting from 5 × 10^18^ cm^−3^ up to 1 × 10^21^ cm^−3^ [45]. The device shows a very small increase in *V_OC_* from 0.598 V (at an emitter doping of 5 × 10^18^ cm^−3^) to 0.668 V (at an emitter doping of 1 × 10^21^ cm^−3^). However, the current density drawn is decreased when the emitter doping increases. This is because the diffusion length in the emitter is decreased by heavy doping [46] and, hence, the recombination rate rises as the doping concentration increases (Figure 8a).

Further, Figure 8b displays the PCE and the fill factor of the cell corresponding to different specified emitter doping concentrations. It is apparent that the maximum efficiency (15.92%) occurs at a doping concentration of 1 × 10^20^ cm^−3^. Additionally, increasing the doping concentration of the emitter results in reducing the *V_ON_* of the backward diode from 0.679 V to 0.062 V, and so the performance of the device as a backward diode will be enhanced, as illustrated in Figure 8c. Hence, there is a trade-off between the efficiency of the solar cell, “the device when applying forward biasing”, and the performance of the backward diode, “the device when applying reverse biasing”. Thus, we selected the optimum emitter doping density that achieves two conditions: maximum permitted PCE and much lower *V_ON_* when compared with the *V_ON_* of the Schottky diode. Referring to Figure 8, we can observe that the maximum permitted PCE occurs at an emitter doping concentration of 1 × 10^20^ cm^−3^, at which the *V_ON_* is 0.123 V.

### 4.3. Impact of BSF Doping Concentration

**Figure 9:**
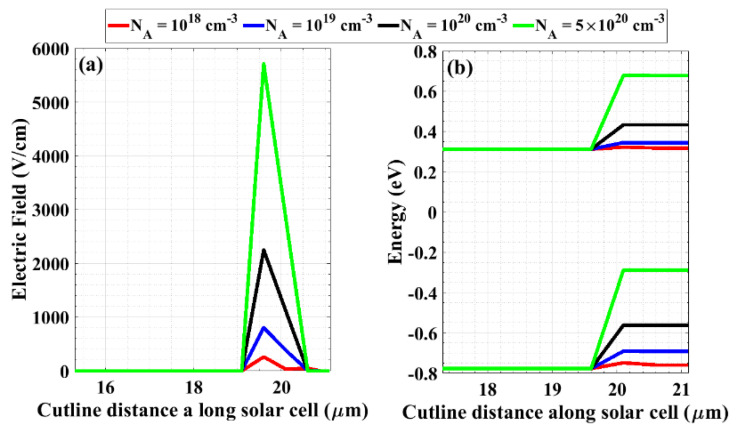
(**a**) Electric field distribution at different BSF doping concentrations near the rear interfaces, and (**b**) an illustration of the energy band diagram near the back interfaces for different values of BSF doping density.

Notably, the BSF layer is a heavily doped layer at the rear side of the solar cell that behaves as a barrier for the transport of minority carriers towards the back surface. At low doping densities, the electric field is weak at the interface resulting in a lower effective collection of photocarriers at the contact. On the other hand, high BSF doping produces a stronger field at the interface. This can be demonstrated by plotting the electric field distribution, as shown in Figure 9a. In addition, the influence of the BSF doping level on the energy band diagram of the proposed structure near the rear interfaces is depicted in Figure 9b. As the BSF doping concentration increases, the barrier for the minority carrier transport towards the back surface also increases. This reduces surface recombination, resulting in higher values of *V_OC_* and *J_SC_*. In this simulation, the BSF doping concentration is changed from 1 × 10^17^ cm^−3^ to 1 × 10^21^ cm^−3^ by keeping the n^++^ emitter doping density constant at 1 × 10^20^ cm^−3^.

**Figure 10:**
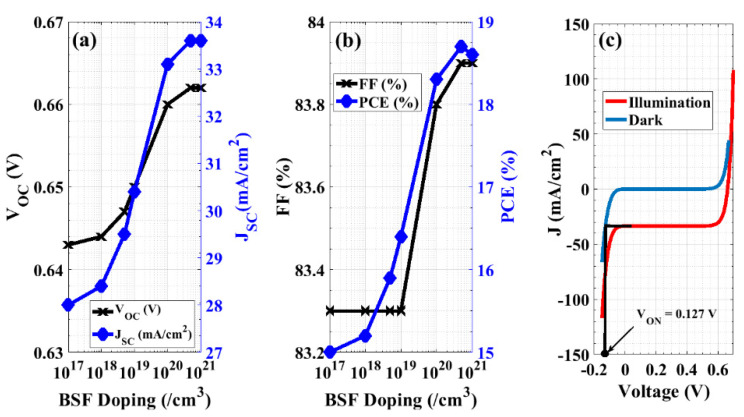
Performance of the modified cell by optimizing the doping density of the BSF layer. (**a**) Open-circuit voltage and short-circuit current density, (**b**) the fill factor and power conversion efficiency, and (**c**) *J-V* characteristics under illumination (red) and in the dark situation (blue) to determine the ON-state voltage of the backward diode.

Moreover, Figure 10a shows the variation in *V_OC_* and *J_SC_* due to the change in the BSF doping density, while Figure 10b indicates the fill factor and PCE as a function of the BSF doping concentration. It can be also observed that there is a quick rise in *J_SC_* and *V_OC_* and, hence, the PCE with an increasing doping concentration up to 5 × 10^20^ cm^−3^, after which saturation occurs. From the *J-V* characteristics demonstrated in Figure 10c, the electrical performance can be extracted as follows: *V_OC_* = 0.662 V, *J_SC_* = 33.58 mA/cm^2^, FF = 83.87 %, and PCE equals 18.65% while keeping the performance as a backward diode approximately with no change (*V_ON_* = 0.127 V).

## 5. Applying Antireflective Coating Layers

One of the remarkable losses in solar cells is the optical loss resulting from surface reflections. The optical losses are translated to an efficiency reduction in the solar cell [56]. Therefore, minimizing the optical losses will significantly result in boosting the efficiency [57]. The antireflective coating has a remarkable positive impact on reducing the reflection and improving the efficiency of solar cells [58,59]. In this section, we will study the influence of distinctive ARC layers on the performance of our self-protected cell.

**Figure 11:**
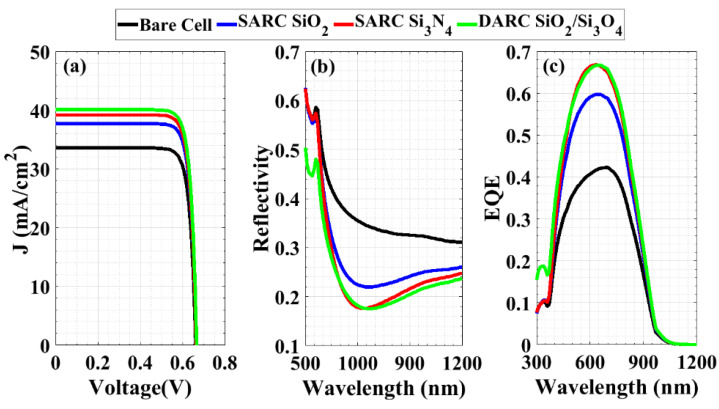
(**a**) *J-V* characteristics of non-coated (bare) and three distinct coated c-Si solar cells, (**b**) reflectivity vs optical wavelength, and (**c**) EQE vs. optical wavelength for different coated and non-coated devices.

The most common coatings used are silicon oxide SiO_x_, silicon nitride Si_3_N_4_, and titanium dioxide TiO_2_, though others are used as well [60,61,62]. Firstly, a silicon oxide single antireflective coating (SARC) with an optimized thickness of 101.351 nm [61] was employed. By using the optimum values for the doping density of both the emitter (1 × 10^20^ cm^−3^) and the BSF layer (5 × 10^20^ cm^−3^), it results in increasing the *J_SC_* to 37.73 mA/cm^2^ and the *V_OC_* to 0.665 V. Utilizing the SARC of silicon oxide increases the PCE of the proposed cell to 21.05%. Similarly, silicon nitride, Si_3_N_4_ SARC, is designed for a 600 nm wavelength and a 74.257 nm thickness. This design shows the lowest reflectance [61], which in turn increases the conversion efficiency to 21.87% due to increasing the current density. Secondly, we designed the cell using the optimized structure with double ARC layers (DARC). The DARC layer’s refractive index, thickness, and reflectivity follow a complex equation [63]. This also implies that optimum surface passivation lowers the thickness of both the SiO_2_ and Si_3_N_4_ layers. The optimum power conversion efficiency of 22.43% was accomplished with a 57-nm-thick SiO_2_ layer on a 58-nm-thick Si_3_N_4_ ARC. As expected, the *J_SC_* density continued to increase to 40.09 mA/cm^2^. Figure 11a displays the *J-V* characteristics for the bare cell (in black) and three distinct coated proposed thin c-Si solar cells, illustrating the effect of the ARC layers on increasing the short-circuit current density.

Furthermore, Figure 11b,c illustrates the dependency of the available photocurrent and EQE on the wavelength of the incident radiation for different ARC schemes, as well as non-coated (bare) devices. Clearly, the ARC layers cause less optical reflectance and thus more available photocurrent (as depicted in Figure 11a). The dependency of the available photocurrent on the optical wavelength directly reflects on the EQE spectrum, as the EQE is the ratio of available and source photocurrents. Overall, Figure 11c shows that after utilizing ARC, the EQE was significantly enhanced. This rise is because of the decline in reflection when employing ARC. Comparing the silicon nitride and silicon oxide, the device with silicon nitride as an ARC layer generated more available photocurrent. However, the reflectance will be minimized when using DARC layers, thereby generating more photocurrent, which in turn increases the short-circuit current (see Figure 11a). Increasing the short-circuit current results in an increase in the PCE.

**Figure 12:**
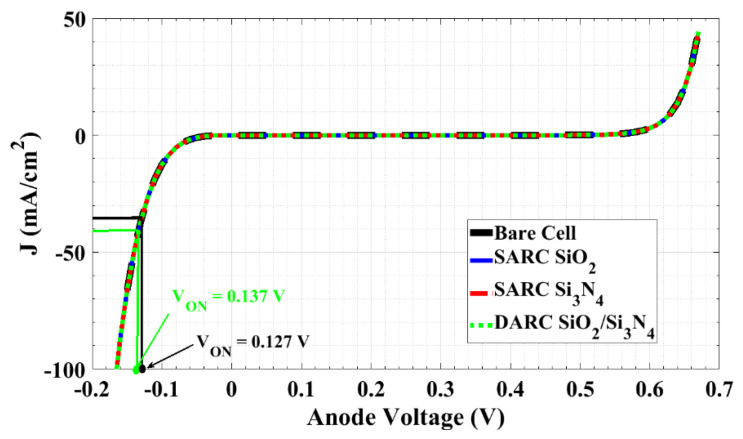
Dark *J-V* Characteristics for bare cell, SARC of silicon oxide, SARC of silicon nitride, and DARC of silicon oxide/silicon nitride.

Moreover, the *J-V* characteristics of the enhanced self-protected thin film cell at reverse biasing utilizing SARC or DARC are demonstrated in Figure 12 for dark conditions. As can be inferred from the figure, the type of antireflection coating has a minor effect on the device’s ON-state voltage as it increases from 0.127 V (bare cell) to just 0.137 V. This can be attributed to the minor voltage changes according to the current, i.e., the low impedance at the reverse bias. Although using ARC increases *J_SC_*, the value of *V_ON_* increases to levels just a little higher than those of the bare cells. Hence, our optimized self-protected thin film c-Si solar cell achieves two important criteria: higher efficiency at forward operation (generating power) and a rapid response to protect the individual cell from high reverse current.

Table 3 summarizes the PV performance metrics, such as the open-circuit voltage, short-circuit current density, fill factor, power conversion efficiency, and the ON-state voltage for the bare cell, the cell with a silicon oxide SARC layer, the cell with a silicon nitride SARC layer, and the SiO_2_/Si_3_N_4_ double ARC layers cell. These performance parameters are compared with the performance of a conventional c-Si solar cell employing a bypass diode, as published in the literature [64]. The comparison illustrates a significant improvement in the ON-state voltage. In addition, the bypass diode is applied for a string of cells [64], while our proposed model is self-protected for each independent cell. Therefore, the power dissipation in our proposed model is expected to be much lower than the power dissipation when using the bypass diode. Finally, some research studies investigated the smart bypass model [54] to improve the ON-state voltage; however, it came with design complexity and cost. The smart bypass model was applied for three series cells.

## 6. Conclusions

In this current study, we presented a state-of-the-art self-protected thin film c-Si solar cell against reverse currents by introducing a heavily doped layer sandwiched between the n-type emitter and the p-type base of the device. The proposed structure showed a significant performance under different biasing conditions. Contrary to the conventional structure that operated as a solar cell, the proposed structure operated as a regular solar cell when applying forward biasing, while it operated as a backward diode by the application of reverse biasing.

We performed comprehensive TCAD simulations to design and evaluate the proposed structure performance. The introduction of the RCL has demonstrated that applying a reverse bias produces a considerably high reverse current within a low ON-state voltage. That is, the cell can protect itself against reverse biasing without adding extra costs compared with the available protection circuit in the market. In addition, the ON-state voltage of the novel device is lower than its candidate for the Schottky bypass diode. Thus, the novel device can be effective to mitigate the impact of overheating or hot spots. The influence of various parameters, such as the doping concentration of the emitter and the BSF layer, on the performance of the novel structure was investigated. After the series of optimization steps, the performance parameters record the following: *V_OC_* = 0.662 V, *J_SC_* = 33.58 mA/cm^2^, FF = 83.87%, and the PCE equals 18.65%, while the ON-state voltage equals 0.127 V.

Additionally, the impact of different single-layer ARCs and a double-layer ARC has been discussed. Applying SiO_2_ and Si_3_N_4_ as antireflection coating layers results in increasing the power conversion efficiency to 21.05% and 21.87%, respectively, while the application of the double ARC layer of SiO_2_/Si_3_N_4_ shows a remarkable rise in the short-circuit current that improves the conversion efficiency to 22.43% as well, keeping the ON-state voltage to be as low as 0.138 V.

## Figures and Tables

**Table 2:** Comparison between the PV parameters of the initial novel design and the normal structure.

	*V_OC_* [V]	*J_SC_* [mA/cm^2^]	*FF* [%]	*PCE* [%]	*P_max_* [mW/cm^2^]
Novel Design	0.667	25.88	83.67	14.45	14.46
Normal Design	0.667	25.81	83.96	14.46	14.47

**Table 3:** Comparison between the associated performance parameters of bare and different coated thin-film c-Si solar cells after enhancement and performance of the initial design.

	*V_OC_* (V)	*J_SC_* (mA/cm^2^)	*FF* (%)	*PCE* (%)	*V_ON_* (V)
Bare Cell	0.662	33.58	83.87	18.65	0.127
SARC of SiOx	0.665	37.73	83.90	21.05	0.131
SARC of Si_3_N_4_	0.665	39.24	83.83	21.87	0.137
DARC	0.667	40.09	83.89	22.43	0.138
Design of [64]	0.646	38.44	80.49	19.95	0.4

## Data Availability

Not applicable.

## References

[1] Andreani L.C., Bozzola A., Kowalczewski P., Liscidini M., Redorici L. (2019). Silicon Solar Cells: Toward the Efficiency Limits. Adv. Phys. X.

[2] Bhattacharya S., John S. (2019). Beyond 30% Conversion Efficiency in Silicon Solar Cells: A Numerical Demonstration. Sci. Rep..

[3] Okil M., Salem M.S., Abdolkader T.M., Shaker A. (2022). From Crystalline to Low-Cost Silicon-Based Solar Cells: A Review. Silicon.

[4] Ushasree P.M., Bora B., Gibson E.A. (2019). Silicon Solar Cells. Solar Energy Capture Materials.

[5] Battaglia C., Cuevas A., de Wolf S. (2016). High-Efficiency Crystalline Silicon Solar Cells: Status and Perspectives. Energy Environ. Sci..

[6] Hwang I., Um H.-D., Kim B.-S., Wober M., Seo K. (2018). Flexible Crystalline Silicon Radial Junction Photovoltaics with Vertically Aligned Tapered Microwires. Energy Environ. Sci..

[7] Hadibrata W., Es F., Yerci S., Turan R. (2018). Ultrathin Si Solar Cell with Nanostructured Light Trapping by Metal Assisted Etching. Sol. Energy Mater. Sol. Cells.

[8] Wang S., Weil B.D., Li Y., Wang K.X., Garnett E., Fan S., Cui Y. (2013). Large-Area Free-Standing Ultrathin Single-Crystal Silicon as Processable Materials. Nano Lett..

[9] Zhou S., Yang Z., Gao P., Li X., Yang X., Wang D., He J., Ying Z., Ye J. (2016). Wafer-Scale Integration of Inverted Nanopyramid Arrays for Advanced Light Trapping in Crystalline Silicon Thin Film Solar Cells. Nanoscale Res. Lett..

[10] Yu K.J., Gao L., Park J.S., Lee Y.R., Corcoran C.J., Nuzzo R.G., Chanda D., Rogers J.A. (2013). Light Trapping in Ultrathin Monocrystalline Silicon Solar Cells. Adv. Energy Mater..

[11] Pagliaro M., Ciriminna R., Palmisano G. (2008). Flexible Solar Cells. ChemSusChem.

[12] Wang K.X., Yu Z., Liu V., Cui Y., Fan S. (2012). Absorption Enhancement in Ultrathin Crystalline Silicon Solar Cells with Antireflection and Light-Trapping Nanocone Gratings. Nano Lett..

[13] Hwang I., Jeong Y., Shiratori Y., Park J., Miyajima S., Yoon I., Seo K. (2020). Effective Photon Management of Non-Surface-Textured Flexible Thin Crystalline Silicon Solar Cells. Cell Rep. Phys. Sci..

[14] Yamamoto K., Nakajima A., Yoshimi M., Sawada T., Fukuda S., Suezaki T., Ichikawa M., Koi Y., Goto M., Meguro T. (2004). A High Efficiency Thin Film Silicon Solar Cell and Module. Sol. Energy.

[15] Sharma M., Pudasaini P.R., Ruiz-Zepeda F., Elam D., Ayon A.A. (2014). Ultrathin, Flexible Organic–Inorganic Hybrid Solar Cells Based on Silicon Nanowires and PEDOT:PSS. ACS Appl. Mater. Interfaces.

[16] Ko M.-D., Rim T., Kim K., Meyyappan M., Baek C.-K. (2015). High Efficiency Silicon Solar Cell Based on Asymmetric Nanowire. Sci. Rep..

[17] Mi Y., Wen L., Xu R., Wang Z., Cao D., Fang Y., Lei Y. (2016). Constructing a AZO/TiO_2_ Core/Shell Nanocone Array with Uniformly Dispersed Au NPs for Enhancing Photoelectrochemical Water Splitting. Adv. Energy Mater..

[18] Lin C.-C., Chuang Y.-J., Sun W.-H., Cheng C., Chen Y.-T., Chen Z.-L., Chien C.-H., Ko F.-H. (2015). Ultrathin Single-Crystalline Silicon Solar Cells for Mechanically Flexible and Optimal Surface Morphology Designs. Microelectron. Eng..

[19] Lin Q., Sarkar D., Lin Y., Yeung M., Blankemeier L., Hazra J., Wang W., Niu S., Ravichandran J., Fan Z. (2017). Scalable Indium Phosphide Thin-Film Nanophotonics Platform for Photovoltaic and Photoelectrochemical Devices. ACS Nano.

[20] Branham M.S., Hsu W.-C., Yerci S., Loomis J., Boriskina S.V., Hoard B.R., Han S.E., Chen G. (2015). 15.7% Efficient 10-Μm-Thick Crystalline Silicon Solar Cells Using Periodic Nanostructures. Adv. Mater..

[21] Pathi P., Peer A., Biswas R. (2017). Nano-Photonic Structures for Light Trapping in Ultra-Thin Crystalline Silicon Solar Cells. Nanomaterials.

[22] Baliozian P., Tepner S., Fischer M., Trube J., Herritsch S., Gensowski K., Clement F., Nold S., Preu R. The International Technology Roadmap for Photovoltaics and the Significance of Its Decade-Long Projections. Proceedings of the 37th European Photovoltaic Solar Energy Conference and Exhibition.

[23] Zekry A., Shaker A., Salem M. (2018). Solar Cells and Arrays. Advances in Renewable Energies and Power Technologies.

[24] Oprea S.-V., Bara A., Preotescu D., Elefterescu L. (2019). Photovoltaic Power Plants (PV-PP) Reliability Indicators for Improving Operation and Maintenance Activities. A Case Study of PV-PP Agigea Located in Romania. IEEE Access.

[25] Oh J., Rammohan B., Pavgi A., Tatapudi S., Tamizhmani G., Kelly G., Bolen M. (2018). Reduction of PV Module Temperature Using Thermally Conductive Backsheets. IEEE J. Photovolt..

[26] Chen K., Chen D., Zhu Y., Shen H. (2012). Study of Crystalline Silicon Solar Cells with Integrated Bypass Diodes. Sci. China Technol. Sci..

[27] Dhimish M., Holmes V., Mehrdadi B., Dales M., Mather P. (2017). Detecting Defective Bypass Diodes in Photovoltaic Modules Using Mamdani Fuzzy Logic System. Glob. J. Res. Eng. F Electr. Electron. Eng..

[28] Vieira R., de Araújo F., Dhimish M., Guerra M. (2020). A Comprehensive Review on Bypass Diode Application on Photovoltaic Modules. Energies.

[29] Wolf E.J., Gould I.E., Bliss L.B., Berry J.J., McGehee M.D. (2022). Designing Modules to Prevent Reverse Bias Degradation in Perovskite Solar Cells When Partial Shading Occurs. Sol. RRL.

[30] Daliento S., Mele L., Bobeico E., Lancellotti L., Morvillo P. (2007). Analytical Modelling and Minority Current Measurements for the Determination of the Emitter Surface Recombination Velocity in Silicon Solar Cells. Sol. Energy Mater. Sol. Cells.

[31] Wohlgemuth J., Herrmann W. (2005). Hot Spot Tests for Crystalline Silicon Modules. Proceedings of the Conference Record of the Thirty-First IEEE Photovoltaic Specialists Conference.

[32] Pezeshki Z., Zekry A., Inamuddin Ahamed M.I., Boddula R., Rezakazemi M. (2021). State-of-the-Art and Prospective of Solar Cells. Fundamentals of Solar Cell Design.

[33] Silvestre S., Boronat A., Chouder A. (2009). Study of Bypass Diodes Configuration on PV Modules. Appl. Energy.

[34] Guerriero P., Cennamo P.A., Matacena I., Daliento S. Avoiding the Hot Spot Occurrence in PV Modules. Proceedings of the 2018 IEEE International Conference on Environment and Electrical Engineering and 2018 IEEE Industrial and Commercial Power Systems Europe EEEIC/I&CPS Europe.

[35] Ayache K., Chandra A., Cheriti A. Second Quadrant Electrothermal Characterization of Photovoltaic Cells for Safe Reverse Bias Operation and Improved Shadow Performances. Proceedings of the 2018 IEEE Industry Applications Society Annual Meeting (IAS).

[36] Witteck R., Siebert M., Blankemeyer S., Schulte-Huxel H., Kontges M. (2020). Three Bypass Diodes Architecture at the Limit. IEEE J. Photovolt..

[37] Ghosh S., Yadav V.K., Mukherjee V. (2020). A Novel Hot Spot Mitigation Circuit for Improved Reliability of PV Module. IEEE Trans. Device Mater. Reliab..

[38] Dhimish M., Holmes V., Mather P., Sibley M. (2018). Novel Hot Spot Mitigation Technique to Enhance Photovoltaic Solar Panels Output Power Performance. Sol. Energy Mater. Sol. Cells.

[39] Karmakar B.K., Pradhan A.K. (2020). Detection and Classification of Faults in Solar PV Array Using Thevenin Equivalent Resistance. IEEE J. Photovolt..

[40] Haug A. (1978). Auger Coefficients for Highly Doped and Highly Excited Semiconductors. Solid State Commun..

[41] Slotboom J.W. (1977). The Pn-Product in Silicon. Solid State Electron..

[42] (2016). Silvaco International ATLAS User’s Manual, Device Simulation Software; Silvaco, Inc. https://silvaco.com/products/tcad/device_simulation/atlas/atlas.html.

[43] N&K Database. http://www.sspectra.com/sopra.html.

[44] Zekry A., Gerlach W. (1988). Reduction of the Current Gain of the N-p-n Transistor Component of a Thyristor Due to the Doping Concentration of the p-Base. IEEE Trans. Electron. Devices.

[45] Salem M.S., Zekry A., Shaker A., Abouelatta M. Design and Simulation of Proposed Low Cost Solar Cell Structures Based on Heavily Doped Silicon Wafers. Proceedings of the 2016 IEEE 43rd Photovoltaic Specialists Conference (PVSC).

[46] Wolf M. (1986). The Influence of Heavy Doping Effects on Silicon Solar Cell Performance. Sol. Cells.

[47] Procel P., Maccaronio V., Crupi F., Cocorullo G., Zanuccoli M., Magnone P., Fiegna C. (2014). Analysis of the Impact of Doping Levels on Performance of Back Contact-Back Junction Solar Cells. Energy Procedia.

[48] Barman B., Kalita P.K. (2021). Influence of Back Surface Field Layer on Enhancing the Efficiency of CIGS Solar Cell. Sol. Energy.

[49] Fossum J.G. (1977). Physical Operation of Back-Surface-Field Silicon Solar Cells. IEEE Trans. Electron. Devices.

[50] von Roos O. (1978). A Simple Theory of Back Surface Field (BSF) Solar Cells. J. Appl. Phys..

[51] Yang H., Wang H., Wang M. (2012). Investigation of the Relationship between Reverse Current of Crystalline Silicon Solar Cells and Conduction of Bypass Diode. Int. J. Photoenergy.

[52] Breitenstein O. (2013). Understanding the Current-Voltage Characteristics of Industrial Crystalline Silicon Solar Cells by Considering Inhomogeneous Current Distributions. Opto Electron. Rev..

[53] Yang Y., Kim K.A., Blaabjerg F., Sangwongwanich A. (2019). PV System Modeling, Monitoring, and Diagnosis. Advances in Grid-Connected Photovoltaic Power Conversion Systems.

[54] Pannebakker B.B., de Waal A.C., van Sark W.G.J.H.M. (2017). Photovoltaics in the Shade: One Bypass Diode per Solar Cell Revisited. Prog. Photovolt. Res. Appl..

[55] Pennisi S. (2011). Low-Power Cool Bypass Switch for Hot Spot Prevention in Photovoltaic Panels. ETRI J..

[56] Fahim N., Ouyang Z., Zhang Y., Jia B., Shi Z., Gu M. (2012). Efficiency Enhancement of Screen-Printed Multicrystalline Silicon Solar Cells by Integrating Gold Nanoparticles via a Dip Coating Process. Opt. Mater. Express.

[57] Park H., Kwon S., Lee J.S., Lim H.J., Yoon S., Kim D. (2009). Improvement on Surface Texturing of Single Crystalline Silicon for Solar Cells by Saw-Damage Etching Using an Acidic Solution. Sol. Energy Mater. Sol. Cells.

[58] Hiller J., Mendelsohn J.D., Rubner M.F. (2002). Reversibly Erasable Nanoporous Anti-Reflection Coatings from Polyelectrolyte Multilayers. Nat. Mater..

[59] Kosyachenko L.A. (2011). Solar Cells—Thin-Film Technologies.

[60] Abdullah L., Abdullah H., Shila Z.M., Hannan M.A. (2010). Modelling and Simulation of SiO_2_/Si_3_N_4_ as Anti-Reflecting Coating for Silicon Solar Cell by Using Silvaco Software. World Appl. Sci. J..

[61] Hashmi G., Rashid M.J., Mahmood Z.H., Hoq M., Rahman M.H. (2018). Investigation of the Impact of Different ARC Layers Using PC1D Simulation: Application to Crystalline Silicon Solar Cells. J. Theor. Appl. Phys..

[62] Sahouane N., Zerga A. (2014). Optimization of Antireflection Multilayer for Industrial Crystalline Silicon Solar Cells. Energy Procedia.

[63] Double Layer Anti Reflection Coatings PVEducation. https://www.pveducation.org/pvcdrom/design-of-silicon-cells/double-layer-anti-reflection-coatings..

[64] Hanifi H., Pander M., Jaeckel B., Schneider J., Bakhtiari A., Maier W. (2019). A Novel Electrical Approach to Protect PV Modules under Various Partial Shading Situations. Sol. Energy.

